# Evaluating the effect of optimal zinc amino-acid complex supplementation in laying pullets on performance and zinc retention

**DOI:** 10.1371/journal.pone.0311813

**Published:** 2024-10-17

**Authors:** Gabriela Duarte Silva, Carlos Bôa-Viagem Rabello, Jamille Sheila da Silva Wanderley, Katariny Lima de Abreu, Lilian Francisco Arantes de Sousa, Rafael Victor Nunes Lima, Fabiano Séllos Costa, Miriam Nogueira Teixeira, Marcos José Batista dos Santos, Alba K. Fireman

**Affiliations:** 1 Universidade Federal Rural de Pernambuco, Recife, Pernambuco, Brazil; 2 Zinpro Corporation, Eden Prairie, Minnesota, United States of America; Zagazig University Faculty of Agriculture, EGYPT

## Abstract

This study investigated the optimal dietary intake of zinc amino acid complex (Zn-AAC) for white-layer pullets, focusing on their productive performance, biochemical profile, organ biometry, and body zinc retention. The study involved 360 Dekalb White pullets (average weight: 433 ± 4.42 g) aged from 6 to 16 weeks and distributed into 6 treatments with 6 replications each. The Zn-AAC inclusion levels ranged from 5 to 75 mg kg^-1^. Zinc intake was modeled using a nonlinear equation, Y = ∝ *1- e^-βX^, where α is the maximum response, β is the rate at which the response approaches the maximum (P < 0.05). The Zn-AAC supplementation had significant effects on average daily gain (ADG), average daily feed intake (ADFI), and feed convention ratio (FCR) (*P* < 0.01). Optimal intake levels of Zn-AAC were estimated at 0.234, 0.340, and 0.315 mg bird^-1^ day^-1^ (5.42, 7.87, and 7.30 mg kg^-1^) for ADG, ADFI, and FCR, respectively. The Zn-AAC supplementation affected the Zn body retention in pullets *(P* < 0.01), with an optimal ingestion at 1.86 mg bird ^-1^ day^-1^, corresponding to a dietary supplementation of 43.10 mg kg^-1^. Additionally, supplementation affected alkaline phosphatase (ALP) activity *(P* < 0.01) without significant changes in aspartate aminotransferase, albumin, and globulin levels. The optimal Zn-AAC intake level for ALP activity was 1.45 mg bird ^-1^ day^-1^, corresponding to dietary supplementation of 33.60 mg kg^-1^. Based on Zn body retention, we recommend up to 1.86 mg bird ^-1^ day^-1^ of Zn-AAC, which is equivalent to 43.10 mg kg^-1^.

## Introduction

Zinc (Zn) is an essential trace mineral in poultry diets and participates in many metabolic pathways necessary for animal growth and life. It acts as an enzymatic co-factor and transcription factor, participating in various biochemical processes [1]. Supplements composed of Zn complexed to amino acids enhance intestinal absorption by animals since it is absorbed intact by the intestinal mucosa [2] when using intestinal amino acid transporters [1]. Suh improvements prevent dissociation and interactions with other nutrients and antinutritional factors, such as phytic acid [1].

Current research has shown that Zn bound to organic sources influences productive performance, nutrient retention in tissues, and egg quality, while also reducing the environmental impact caused by excessive Zn excretion [3–6]. Zinc performs antioxidant actions [7] and increases the immune capacity [8,9]. Zn sources with increased bioavailability in the animal organism explain the egg industry finance investment, as they help avoid losses related to the mortality and morbidity of hens. Moreover, they reduce egg losses caused by bad shell quality [10].

Despite many studies about Zn organic sources for laying hens and broilers had already been conducted, no research determined the Zn amino acid complex (Zn-AAC) requirements for laying pullets consuming diets containing phytase, where all other trace minerals (except iodine) were also supplemented as amino acid metals. Furthermore, laying pullets’ Zn-AAC requirements may differ from mature laying hens or broilers due to their distinct physiological conditions and growth patterns. During the raising phase, pullets undergo significant skeletal development and have a lower growth rate, which may alter their Zn requirements [11]. Understanding these individual requirements is critical for maximizing pullet development and preparing them for the laying cycle demands. Due to the interactions between inorganic minerals in the gastrointestinal tract and the low absorption rate [1], adding other inorganic mineral sources could harm determining optimal trace mineral levels. This issue highlights the necessity for trials including all trace minerals as complex minerals in diets when determining Zn-AAC requirements.

According to the NRC [12], Zn recommendations for laying hens are 35 mg kg^-1^ for inorganic sources. These recommendations are based on studies conducted with broiler chickens. Abranches et al. [13] recommended 33.08 mg kg^-1^ of Zn chelated to an organic source for laying pullets, representing a 55% reduction in the recommendation of Zn from inorganic sources (73.50 mg kg^-1^). However, such a recommendation should be evaluated with caution as it comes from research conducted with broiler chickens and disregards the specificity of each source type.

In laying hens, bone mineralization requires superior mineral retention to support the construction of future eggshells [14]. In addition, Zn is crucial for the bone structure of laying hens because the process involved in eggshell production places substantial demands on skeletal reserves. During periods of active egg-laying, hens must mobilize large calcium amounts from medullary bone tissue to support daily eggshell calcification [15]. As this unique labile bone tissue undergoes turnover, adequate Zn is essential to facilitate osteoclast and osteoblast activity for bone remodeling [16]. In addition, Zn plays a key role in collagen formation and maturation, providing the organic matrix that facilitates bone mineralization [17]. Without sufficient dietary Zn, this can lead to structural degradation, osteoporosis, and increased fracture risk, severely compromising hen welfare [18]. Therefore, optimizing Zn nutrition is not only critical for the growth and development of the skeletal system but also for maintaining integrity under the significant pressures of the laying cycle.

Furthermore, substantial muscle and fat deposition occurs throughout this phase, preparing the hen for the start of the productive period [11]. Therefore, establishing a Zn-AAC requirement in the rearing phase is crucial, as it influences the expression of genes responsible for growth, controls the animal’s appetite, aids in the absorption of fats, and influences the absorption of fat-soluble vitamins A and E [19]. Furthermore, it plays a role in the body’s defense against auto-oxidation [20].

In contrast, sub-optimal supplementation can lead to lower growth, egg production, and disease susceptibility, which can reduce farm profitability [21]. By finding the optimal Zn-AAC supplementation level, farmers can reduce feed costs, and Zn excretion into the environment, increasing overall flock health and performance [9]. As a result, this research brings an understanding of Zn nutrition in laying pullets, providing insights that can improve industry practices and sustainability. Given the Zn importance for pullets’ performance and bone formation, it was hypothesized that estimating the ideal Zn intake in Zn-AAC form will ensure optimal growth, biochemical profile, and body Zn-retention. Thus, we estimated the supplemental intake of Zn-AAC for laying hens by considering performance, serum biochemistry, and body Zn-retention of laying hen pullets aged from 6 to 13 weeks old.

## Methods

All procedures performed in this study were approved by the Ethics Committee on Animal Use (CEUA) of the Federal Rural University of Pernambuco (UFRPE) under a protocol (n°6000110221). Also, the experiment was carried out in compliance with the European Union directive n°. 2010/63/EU.

### Location, birds, and facilities

The experiment was conducted at the Poultry Research Laboratory (LAPAVE) of the Animal Science Department of the Federal Rural University of Pernambuco (Latitude: 8°01’11.3"S and Longitude: 34°57’14.6"W).

Three hundred sixty laying pullets of the Dekalb White strain (6 to 13 weeks old) were housed in 36 experimental cages equipped with a trough feeder and an automatic drinker with an attached cup. The experimental cages were previously electrostatically painted and measured 50 x 80 x 50 cm, using a density of 140 cm^2^ bird^-1^. Water was supplied ad libitum during the experiment, and feed amounts were adjusted weekly according to nutritional requirements.

Air temperature and humidity were recorded daily using a data logger (HOBO 12–012) located at the shed’s extremities and a digital thermo-hygrometer (Incoterm, model 663.02.0.00) situated in the shed’s middle. The following averages of maximum, minimum, and average temperatures and air humidity were registered: 32.72°C ± 0.92, 25.19°C 0.56, 28.36 ± 0.54, and 72.69% ± 0.86, respectively. The light program was 12 hours of natural light per day

### Experimental design and treatments

Six-week-old laying pullets were distributed in a completely randomized design with 6 treatments and 6 replicates of 10 birds each, according to their average weight (433 ± 4.42 g). The trial period lasted from the 6th to the 13th week of life. Treatments consisted of nutritive and isoenergetic experimental diets, varying only in the inclusion levels of Zn bound to amino acids: 5, 15, 25, 35, 55, and 75 mg kg^-1^. The organic source contained unspecified essential amino acids (Zinpro Corp., Eden Prairie, MN, United States) bound to ions of Zn in a 1:1 ratio.

The micromineral premix consisted of Zn, copper (Cu), manganese (Mn), and iron (Fe) bound to amino acids, selenium (Se) in the form of zinc-L-selenomethionine, and iodine in the form of calcium iodate. The premix was formulated according to the Dekalb White Nutrition Guide (2009), except for Zn which was mixed according to the treatments. Other nutrients and energy were formulated according to Rostagno et al. [22] to meet nutritional requirements for birds in the rearing phase (Table 1).

**Table 1:** Calculated and analyzed composition of the experimental diets.

Ingredients	%		Nutrition Composition	
Corn	68.18	EMA^4^, kcal/kg		2850
Soybean meal	21.44	Crude protein, %		15.14
Limestone	1.62	Crude protein^5^, %		16.51
Bi-calcium phosphate	0.775	Dry matter^5^, %		89.31
Sodium bicarbonate	0.150	Ash^5^, %		13.86
Salt	0.160	Available Phosphorus, %		0.420
DL-methionine 99	0.085	Calcium, %		1.10
Premix mineral^1^	0.200	Sodium, %		0.160
Premix Vitamin^2^	0.150	Digestible lysine, %		0.670
Phytase^3^	0.006	Digestible methionine, %		0.310
Inert	7.24	Methionine + Cystine %		0.570
Total	100.0	Na+K+Cl (Meq) mEq/kg		178.5

^1^Availa Zn: 120 g kg^-1^ of zinc, Availa Mn: 80 g kg^-1^ of manganese, Availa Cu: 100 g kg^-1^ of cupper, Availa Fe:100 g kg^-1^ of iron, Availa Se: 1000 mg kg^-1^ of selenium, 628 g kg^-1^ de Calcium iodate;

^2^ Vitamin A (mín): 9.000.000,00 UI kg^-1^, Vitamin D3 (mín): 2.500.000,00 UI kg^-1^, Vitamin E (mín): 20.000,00 UI kg^-1^, Vitamin K3 (mín): 2.50 g kg^-1^, Vitamin B1 (mín): 2.0 g kg^-1^, Vitamin B2 (min): 6.0 g kg^-1^, Vitamin B6 (mín): 3.0 g kg^-1^, Vitamin B12 (mín): 15.000,00 mg kg^-1^, Niacin (mín): 35.00 g kg^-1^, Folic acid (min): 1.50 g kg^-1^, Pantothenic acid (min): 11.00 g kg^-1^, Biotin (min): 0.10 g kg^-1^;

^3^Phytase (mín) 10.000 FTU g^-1^;

^4^Metabolizable energy;

^5^Values analyzed [23] and calculated in the natural matter.

After preparing the experimental diets, a 500-g aliquot was taken from each ration and frozen at -20°C for subsequent analyses of concentrations of dry matter (AOAC method n°. 930.15, [23]), crude protein (N × 6.25, AOAC method n°. 954.01, [23]) and mineral matter (AOAC method n°. 942.05, [23]).

The experimental diets and water used during the study were analyzed for mineral composition, as shown in Table 2.

**Table 2:** Concentration of Zn, Mn, Cu, Fe, Ca and P in the diets and water used in the experiment.

Diet *	Zn (mg kg^-1^)	Mn (mg kg^-1^)	Cu (mg kg^-1^)	Fe (mg kg^-1^)	Ca (g kg^-1^)	P total (g kg^-1^)
Zn-CAA^1^ – 5	35.04	67.25	8.46	257.4	7.52	5.19
Zn-CAA – 15	50.88	69.34	8.28	355.8	7.16	5.34
Zn-CAA – 25	60.30	68.11	8.24	221.8	7.86	5.81
Zn-CAA – 35	71.76	69.97	8.46	271.5	7.54	5.57
Zn-CAA – 55	82.13	67.63	8.54	232.8	7.20	5.79
Zn-CAA—75	111.9	66.01	8.44	248.8	7.78	5.48
Water*	0.036	0.009	0.012	0.000	16.12	0.000

*Values obtained in ICP-OES; ^1^Zn-AAC: Zinc amino acid complex.

Zn: Zinc; Mn: Manganese; Cu: Copper; Fe: Iron; Ca: Calcium; P total: Total Phosphorus.

### Performance

Production performance comprised the average daily gain (ADG, g bird^-1^ day^-1^), average daily feed intake (ADFI, g bird^-1^ day^-1^), and feed conversion ratio (FCR, g g^-1^). Feed intake and animal weighing were performed weekly.

### Hematological and biochemical profile

Blood samples were taken from the jugular vein of 2 birds aged 12 weeks per replication, with 5 ml of blood collected per bird. The first 5-mL sample was used to analyze the hematological profile. Red blood cells, leukocytes, and platelets were counted in a Neubauer chamber after dilution with Natt-Herrick’s reagent. Hematocrit levels were determined using the microcapillary method. The second blood sample was centrifuged at 3500 to 4000 rpm for 15 min to analyze the biochemical profile. Then, 2 ml of serum was collected using a pipette and stored in a freezer until the analyses of uric acid, aspartate aminotransferase (AST), alkaline phosphatase (ALP), albumin, total proteins, and globulins. Samples were thawed at room temperature (27°C) and processed according to the methodology described in the BIOCLIN^®^ commercial kit and then read on a Bioclin spectrophotometer (Biolisa Reader).

### Assessment of body retention of zinc

Six birds aged 6 weeks old were subjected to the Zn body retention evaluation, marking the trial beginning. Then, one bird from each experimental plot was subjected to this assessment at 13 weeks old, resulting in 36 birds at all. These birds were selected based on their average live weight. At the end of the experimental period, the birds were humanely euthanized by administering carbon dioxide (CO_2_) to induce anesthesia, followed by cervical dislocation following the guidelines of Resolution N°. 1000 from the Brazilian Federal Council of Veterinary Medicine.

### Sample preparation of mineral analysis

#### Body composition samples

One bird aged 13 weeks old was sampled from each experimental plot, resulting in 36 birds. The bird’s carcasses were stored in vacuum-sealed plastic bags following processing. Then, samples were cooked in a vertical autoclave (Phoenix Inc., São Paulo, Brazil) at 127°C and 1 atm pressure for 2:30 h. Afterward, they were homogenized using an industrial blender (Skymsen Inc., Santa Catarina, Brazil) to produce a representative sample aliquot. The initial (IBC) and final (FBC) body composition samples of the chicks were subsequently freeze-dried (method No. 925.09, AOAC [23]) and preserved for subsequent mineral analysis.

## Sample preparation

Precisely 0.5 g of both the body composition and feed samples were weighed after the drying. These samples were then digested in 6 ml of 65% nitric acid (HNO_3_) using a microwave digestion system (Mars Xpress, Cem Corporation). The digestion involved a three-stage heating protocol: in the first stage, samples were subjected to 1,300 W for 10 min at a temperature of 120°C; the second stage involved 1,500 W for 15 min at 170°C; and in the final stage, the conditions were maintained at 1,500 W for 35 min at 170°C. The resulting solution was filtered using blue quantitative filter paper and then diluted to a final volume of 25 ml with deionized water.

## Quantification process

Calcium, P, Zn, Mn, Cu, and Fe were quantified in feed and water samples. Zn was the sole mineral analyzed in body composition samples. The analyses were conducted at the Environmental Soil Chemistry Laboratory of UFRPE using an optical emission spectrophotometer with an inductively coupled plasma source (Optima 7000 DV ICP-OES, PerkinElmer).

## Body retention of zinc

The Zn body retention (BRZn) was calculated using the following formula:

BRZn = (final carcass weight × final body Zn content)—(initial carcass weight × initial body Zn content).

### Statistical analysis

Initially, the data were subjected to tests for homogeneity of variances using the Box-Cox test and for the normality of residuals using the Crammer-von Misses test. Subsequently, the data was subjected to a non-linear regression analysis (*P <* 0.05) using the PROC NLIN procedure by the Marquardt method in SAS [24].

The relationship between each variable under study and Zn-CAA intake was modeled using the Mitscherlich mathematical function. By inverting this model, it was possible to estimate the optimal intake levels of Zn-CAA for each variable.

The Mitscherlich model (Mitscherlich, 1909) describes the interaction between the study variable and the intake of Zn-CAA through 2 parameters: maximum potential response (α) and the rate of approach to the maximum (β) response, as defined by the following Eq (1):

$$Y=\propto *1-e^{-\beta X}$$ (1)

Where:

- α represents the maximum response,
- e is the base of the natural logarithm (Euler’s number),
- β is the rate at which the response approaches the maximum
- x is the Zn intake.

By inverting this Eq (2), we can determine the optimal Zn-CAA intake required to achieve the maximum response of the variable:

$$Zn-CAA=-\frac{ln(\frac{Y-\alpha }{\alpha })}{\beta }$$ (2)

Furthermore, by differentiating the Mitscherlich model concerning intake, the marginal efficiency of Zn-CAA based on the maximum response can be calculated, using the Eq (3):

$$\frac{d(\Delta y)}{dx}=\alpha \beta e^{-\beta X}$$ (3)

Where:

- $\frac{d(\Delta y)}{dx}$ represents the rate of change in the response variable concerning changes in Zn intake.

## Results

A significant effect was observed on ADG *(P* < 0.01), ADFI *(P* < 0.01), and FCR *(P* < 0.01) in pullets consuming Zn-AAC (Table 3). The optimal intake of Zn-AAC was estimated at 0.234, 0.340, and 0.315 mg bird^-1^ day^-1^ (5.42, 7.87, and 7.30 mg kg^-1^) for ADG, ADFI, and FCR, respectively.

**Table 3:** Pullets performance (6 to 13 weeks old) fed different levels of zinc amino acid complex (Zn-AAC).

Zn-AAC^1^ (mg bird^-1^ day^-1^)	Zn-AAC (mg kg^-1^)^2^	ADG (g bird^-1^ day^-1^)^3^	ADFI (g bird^-1^ day^-1^)^4^	FCR (g.g^-1^)^5^
0.22	5	11.97	42.30	3.535
0.65	15	11.93	42.90	3.598
1.08	25	11.91	42.92	3.603
1.52	35	12.14	44.31	3.612
2.38	55	12.15	43.65	3.590
3.25	75	11.95	42.82	3.582
Mean	-	12.01	43.15	3.59
*P-value*	-	0.001	0.001	0.001
SEM^6^	-	0.069	0.493	0.003
α^7^	-	12.0191	43.3056	3.5970
β^7^	-	25.6703	17.6338	19.0476

^1^Zn-AAC: **Z**inc amino acid complex intake;

^2^ Zn-AAC supplementations;

^3^ADG – average daily gain;

^4^ADFI – average daily feed intake;

^5^FCR – feed conversion intake;

^6^Standart error mean;

^7^Parameters of the mathematical model; Model: Zn-AAC=α*(1-e(-βx)), α=maximum response, β= is the rate at which the response reaches the response maximum, x=Zinc intake.

**Fig 1:**
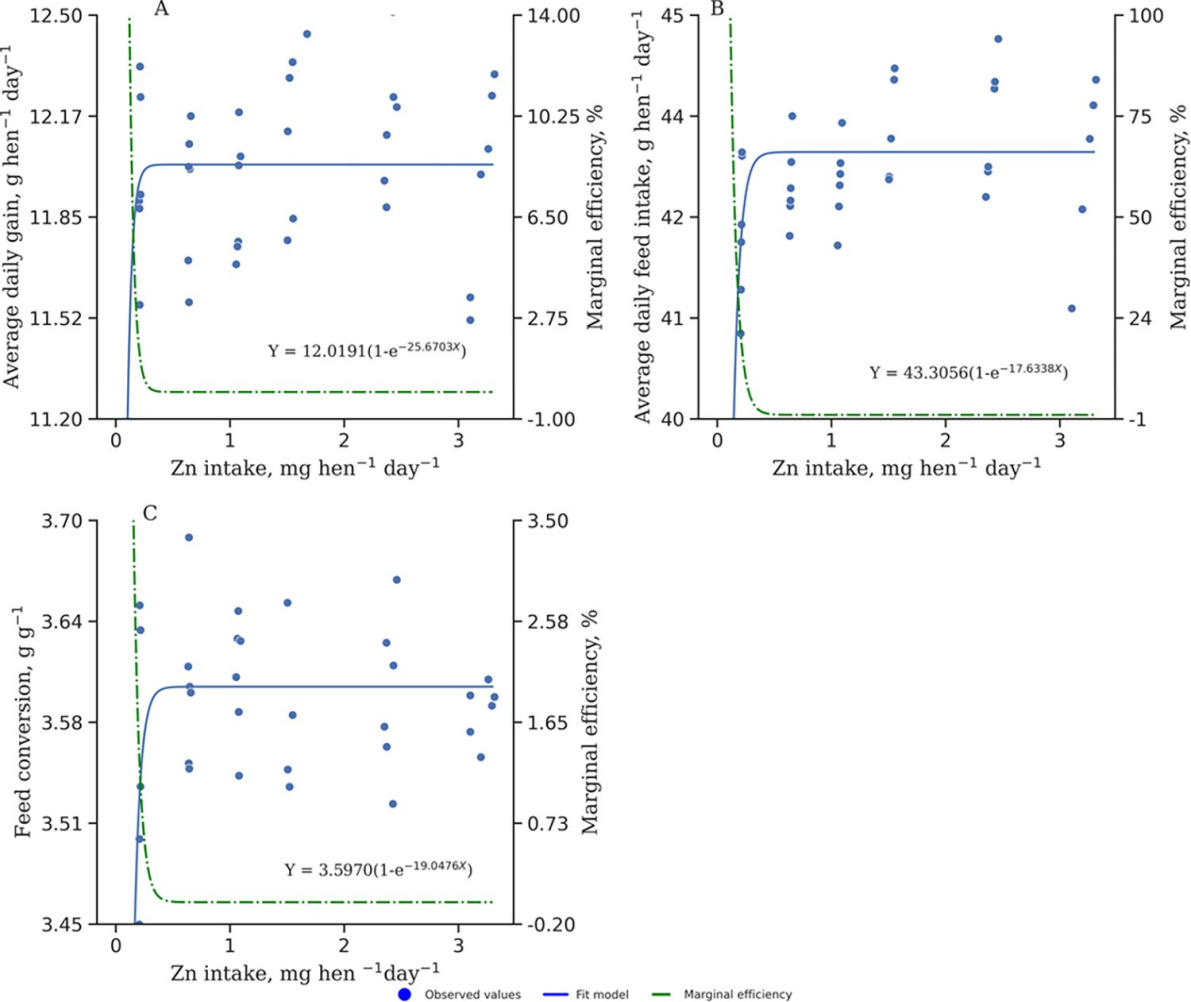
Average daily gain (A), average daily feed intake (B), and feed conversion ratio (C) of zinc amino acid complexed intake of pullets (6 to 13 weeks old). Model: Zn-AAC=α*1-e^(-βx)^, α=maximum response, β= is the rate at which the response reaches the response maximum, x=Zinc intake; Marginal efficiency: αβe-βx.

Fig 1 illustrates the response of BWG, ADFI, and FCR to varying levels of Zn-AAC intake in pullets. The responses to Zn-AAC intake reach a plateau, indicating no further improvement after the maximum response is achieved. This suggests that beyond a certain level of Zn-AAC intake, additional supplementation does not lead to significant improvements in these performance variables. Conversely, the marginal efficiency of Zn-AAC intake declines after reaching the maximum response, demonstrating decreased efficiency of Zn-AAC utilization at higher intake levels.

**Fig 2:**
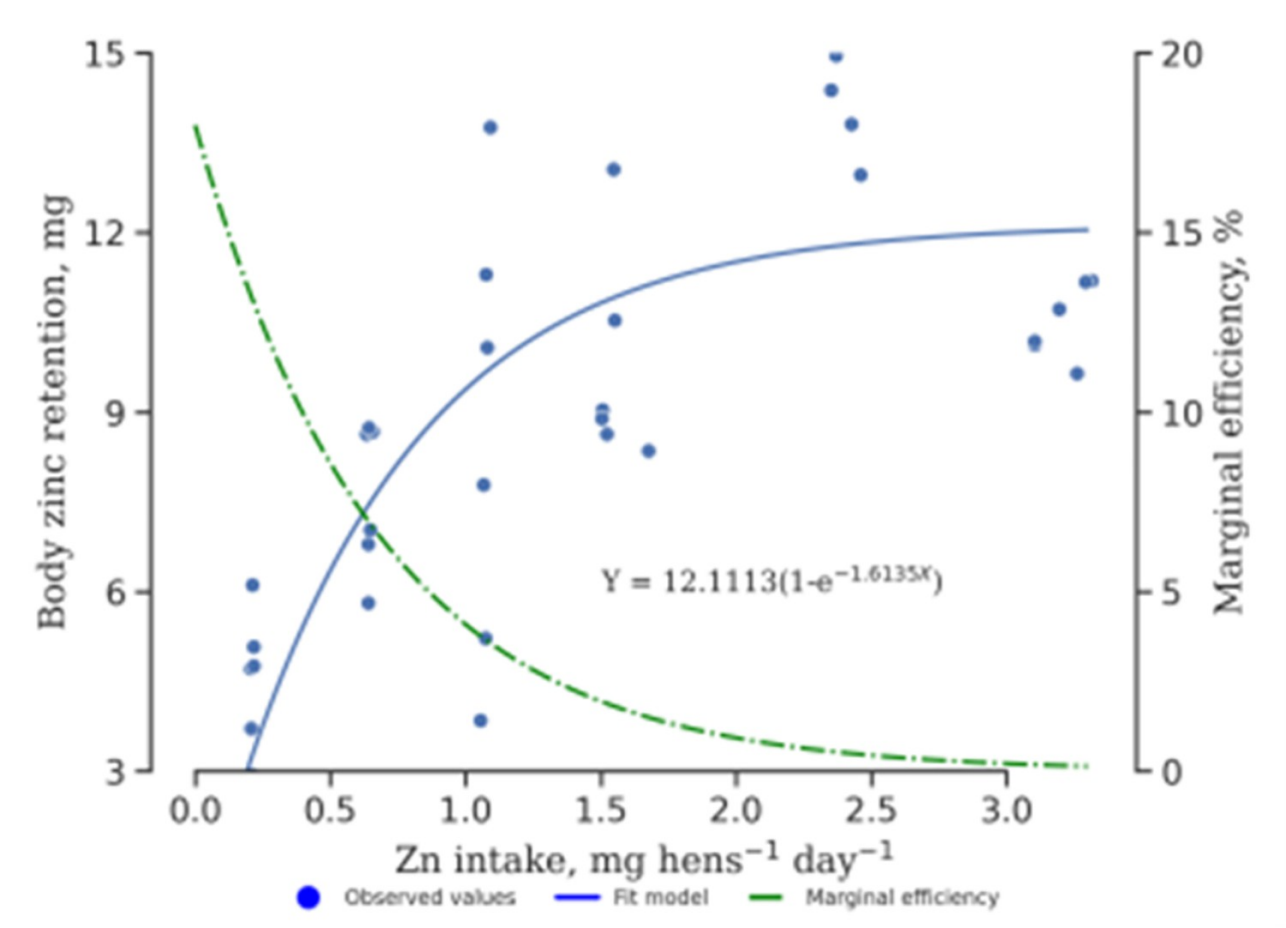
Body retention of zinc amino acid complexed intake of pullets (6 to 13 weeks old). Model: Zn-AAC=α*1-e^-βx^), α=maximum response, β= is the rate at which the response reaches the response maximum, x=Zinc intake; Marginal efficiency: αβe-βx.

Supplemental intake of Zn-AAC *(P* < 0.01) affected the body retention of Zn in pullets (Table 4). An increase in Zn-AAC consumption led to enhanced Zn retention in the bird’s body (BRZn), with a stabilization of retention observed at the maximum consumption level of Zn-AAC (Fig 2). The optimal consumption rate of Zn-AAC was estimated to be 1.86 mg bird ^-1^ day^-1^, corresponding to dietary supplementation of 43.10 mg kg^-1^.

**Table 4:** Body zinc retention in pullets supplemented with zinc amino acid complex (Zn-AAC).

Zn-AAC^1^ (mg bird^-1^ day^-^^1^)	Zn-AAC^2^	FCBW^3^ (g)	FBCZn^4^ (g)	BRZn^5^ (g)
0.22	5	977.0	0.011	4.56
0.65	15	1024	0.013	7.62
1.08	25	1000	0.014	9.63
1.52	35	1047	0.015	9.75
2.38	55	1011	0.020	14.77
3.25	75	1004	0.016	10.51
Mean	-	1010	0.014	9.467
*P-value*	-	0.999	0.999	0.001
SEM^6^	-	1.060	0.0001	0.597
α^7^	-	-	-	12.1113
β^7^	-	-	-	1.6135

^1^Zn-AAC: **Z**inc amino acid complex intake

^2^ Zn-AAC supplementations

^3^Final carcass weight mean

^4^ Final body concentration of Zn

^5^Body zinc retention

^6^Standart error mean

^7^Parameters of the mathematical model; Model: Zn-AAC=α*1-e^-βx^), α=maximum response, β= is the rate at which the response reaches the response maximum, x=Zinc intake; The initial carcass weight was: 433 g; The initial body concentration of Zn: 0.013 g.

**Fig 3:**
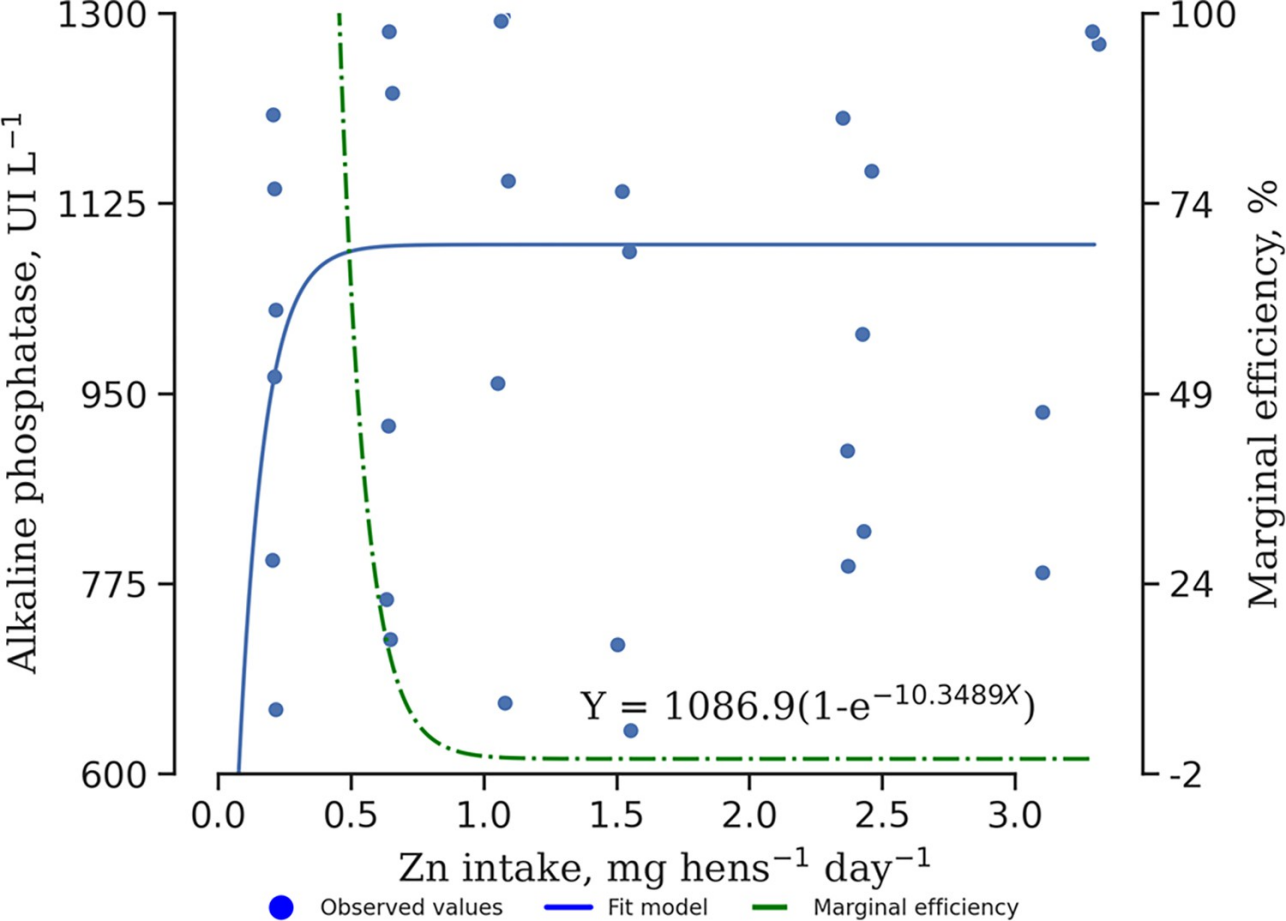
Alkaline phosphatase of pullets supplemented with zinc amino acid complex (6 to 13 weeks old). Model: Zn-AAC=α*(1-e(-βx)), α=maximum response, β= is the rate at which the response reaches the response maximum, x=Zinc intake; Marginal efficiency: αβe-βx.

Supplementation with Zn-AAC only affected the birds’ ALP activity *(P* < 0.01). The AST *(P* = 0.26), ALB *(P* = 0.16), and GLOB *(P* = 0.23) were not changed by the Zn-AAC levels (Table 5). The optimal Zn-AAC consumption was determined to be 0.289 mg bird ^-1^ day^-1^, corresponding to dietary supplementation of 6.59 mg kg^-1^. The model also indicated a plateau after the variables’ maximum response followed by a decrease in marginal efficiency as the Zn-AAC intake increased (Fig 3).

**Table 5:** Biochemical profile of pullets from 6 to 13 weeks old supplemented with zinc amino acid complex (Zn-AAC).

Zn-AAC^1^ (mg bird^-1^ day^-1^)	Zn-AAC^2^ (mg kg^-1^)	AST^3^ (UI L^-1^)	ALP^4^ (UI L^-1^)	ALB^5^ (g L^-1^)	GLOB^6^ (g L^-1^)
0.22	5	188.9	968.3	20.70	20.48
0.65	15	200.4	1054	20.33	19.25
1.08	25	191.7	1158	20.25	19.87
1.52	35	199.9	978.7	20.90	22.00
2.38	55	197.9	979.1	20.62	20.53
3.25	75	197.9	1245	20.62	20.53
Mean	-	196.1	1069	20.57	20.44
*P-value*	-	0.260	0.001	0.160	0.230
SEM^7^	-	0.040	0.060	0.060	0.040
α^8^	-	-	1086.9000	-	-
β^8^	-	-	10.3489	-	-

^1^Zn-AAC: **Z**inc amino acid complex intake

^2^ Zn-AAC supplementations

^3^Aspartate aminotransferase

^4^ Alkaline phosphatases

^5^Albumin

^6^Globulin

^7^Standart error mean

^8^Parameters of the mathematical model; Model: Zn-AAC=α*(1-e^(-βx)^), α=maximum response, β= is the rate at which the response reaches the response maximum, x=Zinc intake.

## Discussion

High-production laying hens are typically raised in cage systems, which allows for efficient control and use of rearing space. However, this confinement limits their mobility and natural locomotion, thus suppressing various biological activities [25]. Such restricted movement diminishes muscle hypertrophy and bone calcification, impairing the overall skeletal health of birds [26]. The skeletal system not only provides structural support but also is a crucial mineral reserve for metabolic activities, playing a vital role in skeletal growth and development.

Based on empirical evidence, it is possible to observe that trace mineral supplementation efficiently promotes skeletal formation and enhances bone mineralization in pullets reared in cages [10]. Increased levels of trace minerals in diets have emerged as a key strategy against physical deterioration and losses in bone strength, currently observed in pullets raised in cage systems [14]. Overcrowded cages can lead to abnormal bone formation, changes in feather growth, dermatitis, losses in the fertile eggs’ hatchability, reproductive issues [27], and suppressed immunity [9].

Bone growth reduces during the rearing phase since the organism prioritizes bone remodeling. The cortical skeleton modulation is essential for depositing the required nutrients throughout the complete production cycle [28].

In this study, we found the productive performance of pullets aligned with their genetic lineage. However, they reached maximum performance when supplemented with Zn-AAC from 0.234 to 0.340 mg bird^-1^ day^-1^, corresponding to dietary supplementation of 5.42 to 7.87 mg kg^-1^. When Zn is deficient or unavailable to the animal, growth, feed intake, and overall development are compromised [29]. Zinc plays a key role in protein catabolism, synthesis, and expression regulation [30,31]. The catalytic activity of RNA polymerases is regulated by using Zn, which is involved in protein synthesis and DNA replication [32]. Zinc acts as a structural component in Zn finger proteins and plays a crucial role in gene expression as a key transcription factor [33]. Zinc finger proteins regulate the growth, development, and immune function of poultry by linking to specific DNA sequences of essential genes [34]. In poultry husbandry, transcription factors containing Zn finger proteins regulate gene expression and protein synthesis.

Brito et al. [35] conducted a comparative analysis of various levels of trace minerals in organic chelates’ form in rearing-period pullets aged from 7 to 12 weeks old, contrasting these with a control diet composed of traditional mineral sources, where levels of Zn-bound to organic molecules were from 30 to 60 mg kg^-1^, and the inorganic Zn level was 60 mg kg^-1^. The authors recommended 30 mg kg^-1^ of supplemented Zn, contrasting the optimal level found in this study for the same rearing phase (up to 5.42 mg kg^-1^ of feed of Zn-AAC to maintain optimal ADG, ADFI, and FCR performances). However, these authors did not specify the mineral complex source, which makes comparisons with other trace mineral sources and practical dietary recommendations inappropriate. Moreover, their source did not demonstrate superiority over conventional ones, indicating a comparable response to that of the inorganic source although the trace mineral was bound to an organic molecule.

Birds ingested more feed as the Zn-AAC levels increased. There is evidence that Zn stimulates poultry feed intake. Researchers found that Zn increases feed via vagal stimulation in rats. Ohinata et al., [36] showed that Zn stimulates feed intake in short-term Zn-deficient through the afferent vagus nerve, affecting the central nervous system. Zinc deficiency can lead to decreased feed intake, while supplementation, especially during early deficiency, can stimulate feed consumption via vagal and neurological pathways [37]. This mechanism may explain the observed higher feed intake as Zn-AAC levels increased in our study.

When supplementing complex trace minerals in animal diets, we expect the molecule to be captured and absorbed intact by the intestinal carriers of the chemical ligand. For instance, metals bound to amino acids are expected to utilize amino acid carriers for absorption, thereby remaining stable in the gastrointestinal tract [1]. This mechanism provides high stability against variations in the pH of the gastrointestinal tract and ensures enhanced bioavailability compared with other complex or non-complex sources [38].

Zinc deficiency primarily impacts the intermediate metabolism of animals, which means symptoms may take time to manifest, posing a challenge for timely diagnosis [39]. Relying only on performance-based estimates to support the hypothesis that specific Zn supplementation aligns with the expected metabolic activities of a given genetic line is inadequate. It is imperative to consider additional factors such as biochemical profiles, bone formation, and Zn retention in this context.

Derivative value at any given point of Zn intake indicates how much additional response can be expected from a small increase in Zn intake at that point. Initially, when Zn intake is low, the marginal efficiency is high, indicating that small increases in Zn intake lead to relatively large increases in all evaluated variables. However, as Zn intake increases, the marginal efficiency decreases because of the exponential decay component, resulting in ever lower returns.

Dietary Zn deficiency can impair the enzymes’ metabolism and hormones involved in growth [40], the immune system [41], and bone development, formation, and mineralization [42,43]. When birds’ diets are supplemented with trace minerals sources bound to amino acids and there is no immediate use by the body, part of this element is retained within the body’s amino acid reserve for further use, rather than being excreted immediately. Such reduced immediate excretion can minimize the poultry industry’s economic and environmental impacts.

Zn retention in laying pullets was the variable chosen for determining the optimal Zn-AAC intake aiming to ensure an adequate Zn reserve in the bird’s body. Based on the top response of retained total Zn (BRZn), pullets required an intake of up to 1.86 mg bird ^-1^ day^-1^ of Zn-AAC, which meant a 43.10 mg kg^-1^ supplementation. Such a level corresponds to 72% of the recommended value in this period for inorganic sources (60 mg kg^-1^) according to the Dekalb White Guidelines. Organic sources such as Zn-AAC are more efficiently used in the birds’ bodies, significantly enhancing absorption efficiency.

The enhanced absorption efficiency of Zn-AAC allows pullets to retain more Zn than inorganic sources at a lower intake level. This pattern indicates that increased bioavailability of Zn from organic sources such as Zn-AAC leads to improved Zn status in pullets even when provided at approximately 30% lower than recommended inorganic Zn levels. Our findings show that supplementing layer diets with 43.10 mg kg^-1^ Zn from Zn-AAC during rearing enables optimal Zn retention at the laying start. Such a level can maintain Zn body reserves through the stress of sexual maturity and ensures a Zn requirement during the early egg production stages when the layer metabolism is higher. Ultimately, an intake of 1.86 mg bird ^-1^ day^-1^ from Zn-AAC during rearing appears adequate for preparing birds for the Zn demands of the lay period. As a consequence, the stabilization of Zn-AAC levels in the birds’ bodies above the maximum ingestion level as evidenced by the body’s retention of Zn-AAC, likely reflects the homeostatic regulation of Zn imported by the body. The observed level was 66% higher than the estimated level for maximal productive performance and bone development responses. In addition, Zn is important for maintaining immunological functions in response to health challenges that can occur in the production environment [9]. An adequate Zn intake supports innate and adaptive immune responses, including cellular immunity, antibody production, and cytokine regulation [44].

Animal health status can be evaluated through blood biochemical levels, which in turn can influence physiological and metabolic responses. Serum biochemistry brings responses of enzyme activity and blood metabolites that indicate liver function and bird immune status [43]. In this study, birds fed with the supplemental Zn-AAC remained in thermal comfort conditions and were housed at appropriate population densities to ensure a suitable biochemical profile. The enzymes’ activities and concentrations remained normal according to poultry standards reported by Lumeij [45] and Campbell [46].

Our results confirm that supplementing pullet diets with Zn-AAC can maintain liver metabolism stability and preserve the bird’s immune status even in the presence of phytase. It is important to consider the release of cations when adding complex trace minerals because phytic acid also releases cations and laying hen diets often have phytase in their compositions [47]. As previously mentioned, these cations are ionized and participate in the absorption processes of enterocytes via specific protein transporters [1].

Zinc supplementation is crucial not only for livestock animals but also for individuals undergoing physiological stress [48]. Research focusing on more bioavailable sources of Zn aims to enhance the animal’s response to lower supplementation levels, optimize responses at specific levels, and improve the sustainability of industries producing animal-based inputs.

Medeiros-Ventura et al. [49] reported that Zn-AAC supplementation supports birds’ defense mechanisms, particularly in cold stress conditions. Such support is primarily due to Zn’s vital role in immune resistance, influencing key immunity mediators such as enzymes, thymic peptides, cytokines, and blood metabolites. The Zn role in regulating lymphoid cell proliferation, activation, and apoptosis was highlighted by Richards et al. [50]. The rearing phase is critical for determining the laying hen flock quality. After this phase, the flock’s characteristics, especially bone quality, must align with the expectations for a productive period set by poultry industry technicians.

Jondreville et al. [51] reported that phytase efficiently enhances Zn availability in growing broiler chicks (up to 25 days old) fed corn and soybean-based diets. They estimated that 100 FTU of microbial phytase can release up to 1.0 mg of Zn. Ao et al. [52] observed that the enzyme phytase released an estimated 4.6 mg of Zn at an inclusion level of 500 FTU kg^-1^. In contrast, we used 600 FTU kg^-1^ of phytase. It is important to be cautious when comparing our study with others, especially those involving broilers, because of variations in diet composition and unanalyzed levels of phytic phosphorus. However, if we assume similar levels of phytic phosphorus in the diets, we could hypothesize that including 600 FTU, as we did, might release at least 5.52 mg of Zn. This would support various biological functions in the pullets, along with the supplementation of Zn-AAC.

Our recommendations for Zn supplemental intake were based on many studied variables. The estimated bodily Zn retention boosts confidence in such recommendations, supporting diverse metabolic functions, even those not measured in the study. This approach can ensure optimal performance, bone health, and immune function, and address organic responses even in challenging situations.

## Conclusion

Based on body Zn retention, it is recommended to supplement 1.86 mg bird ^-1^ day^-1^ of Zn-AAC in white pullet diets containing 600 FTU kg^-1^ of phytase besides Mn, Cu, Fe, and Se complexed to amino acids. Such a recommendation equals 43.10 mg kg^-1^ and can ensure optimal productive performance, immune system support, and metabolic processes.

## References

[1] Goff JP. Invited Review: Mineral Absorption Mechanisms, Mineral Interactions That Affect Acid–Base and Antioxidant Status, and Diet Considerations to Improve Mineral Status. J Dairy Sci 2017, 101:2763–2813. Available from: doi: 10.3168/jds.2017-13112 29397180

[2] Sauer AK, Pfaender S, Hagmeyer S, Tarana L, Mattes AK, Briel F, et al. Charac-terization of Zinc Amino Acid Complexes for Zinc Delivery in Vitro Using Caco-2 Cells and Enterocytes from HiPSC. BioMet-als 2017. Available from: doi: 10.1007/s10534-017-0033-y 28717982 PMC5646115

[3] Behjatian Esfahani M, Moravej H, Ghaffarzadeh M, Nehzati Paghaleh GA. Comparison the Zn-Threonine, Zn-Methionine, and Zn Oxide on Performance, Egg Quality, Zn Bioavailability, and Zn Content in Egg and Excreta of Laying Hens. Biol Trace Elem Res 2021, 199, 292–304. Available from: doi: 10.1007/s12011-020-02141-8 32367378

[4] Zhang YN, Zhang HJ, Wang J, Yue HY, et al. Effect of Dietary Supplementation of Organic or Inorganic Zinc on Carbonic Anhydrase Activity in Eggshell Formation and Quality of Aged Laying Hens. Poult Sci 2017, 96, 2176–2183. Available from: doi: 10.3382/ps/pew490 28204703

[5] Min YN, Liu FX, Qi X, Ji S, Ma SX, Liu X, et al. Effects of Methionine Hydroxyl Analog Chelated Zinc on Laying Performance, Eggshell Quality, Eggshell Mineral Deposition, and Activities of Zn-Containing Enzymes in Aged Laying Hens. Poult Sci 2018, 97, 3587–3593. Available from: doi: 10.3382/ps/pey203 29860354

[6] Cufadar Y, Göçmen R, Kanbur G, Yıldırım B. Effects of Dietary Different Levels of Nano, Organic and Inorganic Zinc Sources on Performance, Eggshell Quality, Bone Mechanical Parameters and Mineral Contents of the Tibia, Liver, Serum and Excreta in Laying Hens. Biol Trace Elem Res 2020, 193, 241–251. Available from: doi: 10.1007/s12011-019-01698-3 30941677

[7] Li LL, Gong YJ, Zhan HQ, Zheng YX, Zou XT. Effects of Dietary Zn-Methionine Supplementation on the Laying Performance, Egg Quality, Antioxidant Capacity, and Serum Parameters of Laying Hens. Poult Sci 2019, 98, 923–931. Available from: doi: 10.3382/ps/pey440 30299460

[8] Han Q, Guo Y, Zhang B, Nie W. Effects of Dietary Zinc on Performance, Zinc Transporters Expression, and Immune Response of Aged Laying Hens. Biol Trace Elem Res 2020, 196, 231–242. Available from: doi: 10.1007/s12011-019-01916-y 31773485

[9] Pereira CG, Rabello CBV, Barros MR, Manso HECCC, Santos MJB, Faria AG, et al. Zinc, manganese and copper amino acid complexed in laying hens’ diets affect performance, blood parameters and reproductive organs development. PLoS One 2020, 15:e0239229, 2020. Available from: doi: 10.1371/journal.pone.0239229 33147220 PMC7641365

[10] Santos MJB, Ludke MCMM, Silva LM, Rabello CBV, Barros MRB, Costa FS, et al. Complexed amino acid minerals vs. bis-glycinate bound minerals: Impact on the performance of old laying hens. Anim Nutr 2023, 23: 2405–6545. Available from: doi: 10.1016/j.aninu.2023.11.006 38371472 PMC10874725

[11] Silva EP, Sakomura NK, De Paula Dorigam JC, Malheiros EB, Peruzzi NJ. Adjustment of Growth Parameters for the Major Body Components of Pullets1. Rev Ciên 2016, 47, 572–581. Available from: doi: 10.5935/1806-6690.20160069

[12] NRC—National research council. Nutrient Requirements of Poultry. (9° ed.). Washington: Acad. Press, 1994.

[13] Abranches FF, Teixeira LM, Gomes MS. Suplementação de vitaminas e de microminerais para aves e suínos. In: Horacio Santiago Rostagno e Luiz Fernando Teixeira Albino(org.). Tabelas brasileiras para aves e suínos. Viçosa, 2024.

[14] Medeiros-Ventura WRL, Rabello CBV, Santos MJB, Barros MR, Silva Junior RV, Oliveira HB, et al. The impact of phytase and different levels of supplemental amino acid complexed minerals in diets of older laying hens. Animals 2023, 13: 1–24. Available from: doi: 10.3390/ani13233709 38067060 PMC10705327

[15] Kerschnitzki M, Zander T, Zaslansky P, Fratzl P. Rapid alterations of avian medullary bone material during the daily egg-laying cycle. Bone 2014, 69:109–117. Available from: doi: 10.1016/j.bone.2014.08.019 25204794

[16] Zhang Y, Deng Y, Jin Y, Zhuang Z, Huang X, Li K, et al. Dietary zinc supplementation affects eggshell quality and ultrastructure in commercial laying ducks by influencing calcium metabolism. Poult Sci 2022, 101:101539. Available from: doi: 10.1016/j.psj.2021.101539 34823167 PMC8628011

[17] Seo HJ, Cho YE, Kim T, Shin HI, Kwun IS. Zinc may increase bone formation through stimulation cell proliferation, alkaline phosphatase activity and collagen synthesis in osteoblastic MC3T3-E1 cells. Nutr Res Pract 2010, 5:356–361. Available from: doi: 10.4162/nrp.2010.4.5.356 21103080 PMC2981717

[18] Huang L, Shen J, Feng Y, Li D, Wang W, Yang L, et al. Effect of dietary Zinc level oh egg production performance and eggshell quality characteristics in laying duck breeders in furnished cage system. Biol Trace Elem Res 2020, 196:597–606. Available from: doi: 10.1007/s12011-019-01927-9 31960274

[19] Mayer AN, Vieira SL, Berwanger E, Angel CR, Kindlein L, França I, et al. Zinc requirements of broiler breeder hens. Poult Sci 2019, 98: 1288–1301. Available from: doi: 10.3382/ps/pey451 30329123

[20] Suttle N. Mineral Nutrition of Livestock. 2010. CABI.

[21] Hidayat C, Sumiati, Jayanegara A, Wina E. Effect of Zinc on the immune response and production performance of broilers: a meta-analysis. Ajas 2020, 33: 465–479. Available from: https://doi.org/10.5713%2Fajas.19.0146. 31208174 10.5713/ajas.19.0146 PMC7054626

[22] Rostagno HS. et al. Suplementação de vitaminas e de microminerais para aves e suínos. In: Horacio Santiago Rostagno(org.). Tabelas brasileiras para aves e suínos. Viçosa, 2017.

[23] Association of Official Analytical Chemists [AOAC]. 2000. Official Methods of Analysis. 17ed. AOAC, Arlington, VA, USA.

[24] SAS. SAS/STAT 3.1. User’s Guide. Version 3.1. Cary, NC: SAS, Institute Inc. 2004.

[25] Santos M, Pandorfi H, Rabello C, Silva E, Torres T, Santos P, et al. Performance of Free-Range Chickens Reared in Production Modules Enriched with Shade Net and Perches. Revista Brasileira de Ciência Avícola 2014, 16, 19–27. Available from: doi: 10.1590/S1516-635X2014000100003

[26] Sinclair-Black M, Garcia RA, Ellestad LE. Physiological Regulation of Calcium and Phosphorus Utilization in Laying Hens. Front Physiol 2023, 14. 10.3389/fphys.2023.1112499 PMC9942826 36824471

[27] Byrne L, Murphy RA. Relative Bioavailability of Trace Minerals in Production Animal Nutrition: A Review. Animals 2022, 12, 1981. Available from: doi: 10.3390/ani12151981 35953970 PMC9367456

[28] Dibner JJ, Richards JD, Kitchell ML, Quiroz MA. Metabolic Challenges and Early Bone Development. J Appl Poult Res 2007, 16, 126–137. Available from: doi: 10.1093/japr/16.1.126

[29] Jahanian R, Moghaddam HN, Rezaei A, Haghparast AR. The influence of Dieatary Zinc-Methionine Substitution for Zinc Sulfate on Broiler Chick Performance. J Biol Sci 2008, 8: 321–327. Available from: doi: 10.3923/jbs.2008.321.327

[30] Cousins RJ. Trace element micronutrients. In: Meyers RA (Org.). Molecular Biology and Biotechnology. New York: VCH Publishers, 1995: 898–901.

[31] Cousins RJ. In Present Knowledge. In: Filer LJ, Ziegler EE (Org.). Nutrition, 7^th^ ed. Washington, DC: International Life Sciences Institute-Nutrition Foundation, 1996: 293–306.

[32] Vallee BL; Falchuk KH. The biochemical basis of zinc physiology. Physiol. Rev. 1993, 73: 79–118. doi: 10.1152/physrev.1993.73.1.79 8419966

[33] Klug A, Rhodes D. Zinc fingers: a novel protein fold for nucleic acid recognition. CSHL: Symposia on Quantitative Biology 1987, 52: 473–482. Available from: doi: 10.1101/sqb.1987.052.01.054 3135979

[34] Shankar AH, Prasad AS. Zinc and immune function: the biological basis of altered resistance to infection. AJCN, 1998, 68: 447S–463S. doi: 10.1093/ajcn/68.2.447S 9701160

[35] Brito JÁG, Bertechini AG, Fassani ÉJ, Rodrigues PB, Freitas RTF. Uso de Microminerais Sob a Forma de Complexo Orgânico Em Rações Para Frangas de Reposição No Período de 7 a 12 Semanas de Idade Effects of Feeding Trace Minerals as Organic Complex for Replacement Pullets in the Period from 7 to 12 Weeks Old. R Bras Zootec 2006, 35, 1342–1348.

[36] Ohinata K, Takemoto M, Kawanago M, Fushimi S, Shirakawa H, Goto T, et al. Orally Administered Zinc Increases Food Intake via vagal Stimulation in Rats J Nutri 2009, 139: 611–616. Available from: doi: 10.3945/jn.108.096370 19158231

[37] Zarghi H, Golian A, Hassanabadi A, Khaligh F. Effect of Supplemental Zinc on Performance, Nutrient Digestibility, Jejunum Architecture, and Immune Response in Broiler chickens Fed Wheat-Soy Diets. An Acad Bras Cienc 2022, 94: e20200266. Available from: doi: 10.1590/0001-3765202220200266 35703687

[38] Gao S, Yin T, Xu B, Ma Y, Hu M. Amino Acid Facilitates Absorption of Copper in the Caco-2 Cell Culture Model. Life Sci 2014, 109, 50–56. Available from: doi: 10.1016/j.lfs.2014.05.021 24931904

[39] Duffy R, Yin M, Redding LE. A Review of the Impact of Dietary Zinc on Livestock Health. J. Trace Elem Min 2023, 5, 100085. Available from: doi: 10.1016/j.jtemin.2023.100085

[40] Muszyński S, Tomaszewska E, Kwiecień M, Dobrowolski P, Tomczyk-Warunek A. Subsequent Somatic Axis and Bone Tissue Metabolism Responses to a Low-Zinc Diet with or without Phytase Inclusion in Broiler Chickens. PLoS One 2018, 13, e0191964. Available from: doi: 10.1371/journal.pone.0191964 29373588 PMC5786321

[41] Jarosz L, Marek A, Gradzki Z, Kwiecień M, Zylińska B, Kaczmarek B. Effect of Feed Supplementation with Zinc Glycine Chelate and Zinc Sulfate on Cytokine and Immunoglobulin Gene Expression Profiles in Chicken Intestinal Tissue. Poult Sci 2017, 96, 4224–4235. Available from: doi: 10.3382/ps/pex253 29053834

[42] Kwiecień M, Winiarska-Mieczan A, Milczarek A, Tomaszewska E, Matras J. Effects of Zinc Glycine Chelate on Growth Performance, Carcass Characteristics, Bone Quality, and Mineral Content in Bone of Broiler Chicken. Livest Sci 2016, 191, 43–50. Available from: doi: 10.1016/j.livsci.2016.07.005

[43] Chen NN, Liu B, Xiong PW, Guo Y, He JN, Hou CC, et al. Safety Evaluation of Zinc Methionine in Laying Hens: Effects on Laying Performance, Clinical Blood Parameters, Organ Development, and Histopathology. Poult Sci 2018, 97, 1120–1126. Available from: doi: 10.3382/ps/pex400 29325174

[44] Hidayat C, Sumiati A, Jayanegara Wina E. Supplementation of dietary nano Zn-Phytogenic on performance, antioxidant activity, and population of intestinal pathogenic bacteria in broiler chickens. Trop Anim Sci 2021, 44: 90–99. Available from: doi: 10.5398/tasj.2021.44.1.90

[45] Lumeij JT. Avian clinical biochemistry, In: Kaneko J.J., Harvey J. W. and Bruss M. L., 1324 Eds., Clinical Biochemistry of Domestic Animals, San Diego: Academic Press, 2008 872: 839–1325. 18833729

[46] Campbell TW. Bioquímica clínica das aves, In THRALL M. A.; WEIXER G.; ALISSON R, W.; CAMPBELL T. W. (org.). Hematologia e bioquímica clínica veterinária. São Paulo: Roca, 2015, 1233–1257.

[47] Liu N, Ru YJ. Effect of Phytate and Phytase on the Ileal Flows of Endogenous Minerals and Amino Acids for Growing Broiler Chickens Fed Purified Diets. Anim Feed Sci Technol 2010, 156, 126–130. Available from: doi: 10.1016/j.anifeedsci.2010.01.008

[48] Huang L, Li X, Wang W, Yang L, Zhu Y. The Role of Zinc in Poultry Breeder and Hen Nutrition: An Update. Biol Trace Elem Res 2019, 192, 308–318. Available from: doi: 10.1007/s12011-019-1659-0 30767181

[49] Medeiros-Ventura WRL, Rabello CBV, Barros MR, Silva Junior RV, Oliveira HB, Faria AG, et al. Zinc, Manganese, and Copper Amino Acid Complexes Improve Performance and Bone Characteristics of Layer-Type Chicks under Thermoneutral and Cold Stress Conditions. Poult Sci 2020, 99, 5718–5727. Available from: doi: 10.1016/j.psj.2020.07.022 33142489 PMC7647727

[50] Richards JD, Zhao J, Harrell RJ, Atwell CA, Dibner JJ. Trace Mineral Nutrition in Poultry and Swine. Asian-Australas J Anim Sci 2010, 23, 1527–1534. Available from: doi: 10.5713/ajas.2010.r.07

[51] Jondreville C, Lescoat P, Magnin M, Feuerstein D, Gruenberg B, Nys Y. Sparing effect of microbial phytase on zinc supplementation in maize–soya-bean meal diets for chickens. Animals, 2007. 6: 804–811. Available from: doi: 10.1017/S1751731107000328 22444743

[52] Ao T, Pierce JL, Pescatore AJ, Cantor AH, Dawson KA, Ford MJ, et al. Effects of Organic Zinc and Phytase Supplementation in a Maize–Soybean Meal Diet on the Performance and Tissue Zinc Content of Broiler Chicks. Br Poult Sci 2007, 48, 690–695. Available from: doi: 10.1080/00071660701694072 18085451

